# The Management of Pulmonary Tuberculosis1Read at a joint meeting of the medical societies of Allegany and Cattaraugus N. Y., and McKean, Pa., at Rock City, N. Y., July 17, 1906.

**Published:** 1906-10

**Authors:** J. C. Young

**Affiliations:** Cuba, N. Y.


### The Management of Pulmonary Tuberculosis.[1]

BY J. C. YOUNG, M. D., M. R. C. S., M. R. C. P., of Cuba, N. Y.

Prevention is better than cure. These are words which have been both echoed and scorned by thousands for a long lease of years. But in this age it becomes the physician's duty to prevent as well as cure. While it is not possible to cure every case of tuberculosis even with the best means at our command, yet it does seem possible that with the present available methods every case, if we except those of possible hereditary transmission, could be regarded as a preventable one. I will incidently refer again to the influence of heredity but my remarks will be confined mostly to the treatment of those cases where prevention has had no opportunity, or if so is not fully carried out and therefore not entirely satisfactory.

As we search the records to find the causes of death in mankind we find on an average one word written in every seven as the cause, and that word is tuberculosis. Nor is that all: the loss in wealth to individual possessors of the disease and mankind at large is something wonderfully enormous.

Over and again we have known many persons who have said they would give all their possessions if they could only thereby purchase health. In no disease that we can mention is there any greater sums of money expended for cure than in cases of tuberculosis.

The question is often asked the medical man: "Is consumption ever curable," and I am glad to be able to say that the answers come thick and fast in the affirmative from the best authorities in the civilized world. Entrusted then with a case of tuberculosis the question naturally comes to our mind, is this particular case a curable one? Now it is not right and not enough for us to say "Well if we could place the patient under the care of some one who makes a specialty in this line and who has spent years of time and thought and study in this and kindred diseases then the chances of recovery would indeed be very great," for we know perfectly well that it would be impossible to place under their care

1. Read at a joint meeting of the medical societies of Allegany and Cattaraugus N. Y., and McKean, Pa., at Rock City, N. Y., July 17, 1906.

and observation anything like the number that comes to us for relief.

The question then for me to ask myself and for each one of you to ask yourselves is this: "Can I cure this disease in this particular patient and will I do all in my power to bring about such a result." The result will depend very much upon the earnestness with which both patient and physician enter into the struggle and very much upon the coöperation of both physician and patient. While I know that it is not always best for patients to know all that is known to us about their real condition, yet given a case of tuberculosis after the patient has reached the years of understanding, or later during these years when the real work of life is being done, we should be frank with them and tell them what disease they have, both for their own protection and the protection of those around them and for our reputation.

When a patient, however, once knows he has consumption he must be encouraged to look upon the bright side, and all the hopes that the facts in the case will warrant must be held out to him. To hold out hope, however, when there are no prospects cannot be too strongly condemned. I have often thought that one reason why tubercular patients retain their hold upon life so long is because they are so hopeful and expectant unto the end. Many tubercular patients are making plans and calculations today for the future, when they get well, and it is inadvisable to encourage them in false hopes.

### CLOTHING.

There are four other points I wish to mention briefly. First, of the clothing. If this is left to the patient's ideas, fancy, or judgment it is invariably wrong. These persons reason thus: "I take cold so easily that I cover up very warm. I am wearing January clothing in July and in January I wear nearly double the amount worn by other members of the family." The result is that the body is kept in a constant state of moisture and upon every exertion, exercise being so important to tubercular patients under certain conditions, it brings on a profuse perspiration and it is not uncommon to find the under garments of such patients completely saturated with moisture. Patients therefore should be instructed as to their clothing.

Soft woolen should be worn the year round, the kind which will readily absorb moisture but is not too heavy. It is better to avoid chest protectors but if worn at all they can be so adjusted as to be easily removed when indoors and this should be insisted upon. The outer garments should be good protectors but should be made so they can be removed readily and replaced as occasion requires. Changes in the extremes of weather should ever be met with suitable clothing. Women should be induced to sacrifice their ideas of clothing or fashion for health. In driving or when lying in open air clothing should be worn suitable to protect the person. The tendency to take cold easily which is ever present in tubercular cases is greatly increased by the excessive clothing generally worn. The one thing that the patient is planning to avoid is greatly increased by the means used to prevent it.

### DIET.

All physicians are agreed that one of the most important elements in the treatment of tuberculosis is a good supply of nourishing and easily digested food. Anything which disturbs digestion or lessens the appetite should be prohibited at once, for food is more important than medicines. The patient should have plenty of food placed before him in the most inviting and tempting manner possible. A good cook using proper judgment about the arrangement of the food table is often of more benefit than the physician himself. Meat, including fish, poultry, game, oysters, milk, eggs, fruit, vegetables, breadstuffs with hot soup for dinner is a desirable list from which to select. We can hardly give too much milk; if the patient can digest it three to six pints a day may be allowed. When raw milk cannot be bourne boiled milk or koumiss may be substituted. Forced feeding with a tube is often valuable when other means fail and should always be tried when indicated. I fully concur in the opinion of those who claim the same quantity of food required for a healthy working person is not sufficient for a tubercular patient. Our aim in tubercular cases should be to administer more than is required so that not only the waste may be met with but the tissues built up and the weight increased if possible.

Some kind of hot drink in the morning before arising aids in the expulsion of mucus. The question of stimulants in tubercular disease has often led to sharp discussions and a wide difference of opinion, but it can be safely stated that it is unwise to use alcoholics in incipient cases. If, however, they have the habit persuade them to abstain from it as soon as possible. In far advanced cases where the result is sure to be fatal a glass of hot milk with whisky in it at bed time or during the night will often afford the patient a better nights rest. In this use of alcohol there is no danger of creating the habit. Look well then to the feeding of tubercular patients liberally for it is one of the most important features in the management.

### HYGIENE AND CLIMATE.

Here one might speak beyond the limits of any paper and not exhaust the subject. Want of exercise and lack of fresh air will work alike to the detriment of the wealthiest banker or with the poorest working girl. A sleeping room should be capable of the most thorough ventilation and have at least 1200 cubic feet of air space. Patients should sleep alone. They need all the air the room contains and it should neither be shared by others nor should lights be burned in the sleeping room. Inhalation of impure and confined air is one great source of infection.

I cannot speak of health resorts or different climates here. Unfortunately those who have spent much time in the study of these subjects are not all agreed, and I sometimes think that too much is made of these differences of opinion. All sorts of climates have at times been strongly recommended and all again have been either condemned or belittled. Recently some good authorities claim that climate has nothing at all to do with results but that they depend upon other measures. The writer believes that while he is speaking his own sentiments he is also that of others who have given the subject careful attention, in asserting that the climate which affords the greatest facilities for spending the largest amount of time in the open air and where it is neither excessively hot or moist is the one most likely to lead to the best results.

The patient should become so accustomed to out-door life that he can remain in the open even during the most unpromising weather. Out door games, exercises, and amusements should be cultivated, and occupation with the poor and habits with the rich must be given up if they interfere with out door life and exercise. Where a cure has followed a change of climate or a change in occupation, probably the factor which deserves the most credit has been the open air life which either from disposition or environment has been forced upon the patient. Impress upon the patient that climate or a health resort is nothing if he maintains sedentary habits, late hours, or a life of either fashion or pleasure with a dislike to open air.

The open air treatment has become almost a household word, yet people very rarely understand its real meaning, and it will be sometime before all can be educated to know that it means something more than to open a door or a window for a few moments and we must do all we can to overcome such objections.

If I were asked which is best for a tubercular case, summer heat or winter cold, I would say by all means winter cold if open air is coupled with it. Pure cold air quiets cough, lessens fever, and increases the appetite. Dry cold air expands as it is warmed in the lungs and is beneficial in that way. Fresh air is of equal importance to food and perhaps to the recognition of this fact is due the improved methods in the management of tubercular cases. Probably Dr. Henry McCormac, who did so much with voice and pen, will ever be mentioned as the pioneer who pointed out the evils attending the rebreathing of expired air. From this has sprung up ideas in regard to the open air treatment which have done more to prevent or cure tuberculosis than any other fact that could be mentioned.

The open air treatment then cannot be spoken of in too glowing colors. Instead of keeping these patients carefully protected from fresh air as was formerly the case they should be kept summer and winter, rain or snow, in the open air. Of course patients should be under cover and protected from storms but the most free ventilation should be carried out in the daily life. The open air treatment does not mean that a patient must exercise sufficiently to exhaust his strength. While the temperature is at any time above 100° it is better for him to be kept at rest.

Sanitariums and tent colonies for the treatment of tubercular cases are of recent origin. The first one in America was erected 20 years ago with capacity for nine patients. Now there are between 140 and 150 for the same purpose. That of itself speaks volumes to be placed on the credit side of the ledger to those institutions. I cannot enter into details but there is no doubt that this has given the best results of any line of treatment ever proposed. The wards of a general hospital should no longer be used for tubercular cases as the other inmates are thus placed in an atmosphere of danger.

But, unfortunately, very many cases cannot avail themselves of a change of climate or a health resort or a sanitarium or tent colony life. It is a fact that 25% of the families in the United States are living on $400 a year or less. Now, from out of these homes and among these families quite a large percentage of tubercular patients come, hence they must remain under the care and observation of their family physician until relieved by art or released by death. States should take cognisance of this fact, for a nation's humanity and wisdom is nowhere better shown than in the care and safety it gives its helpless and dependent people.

But under present conditions we should do all for these patients that we can, and let it not be said by those who follow us that our eyes were too dim or our enthusiasm too cold, to recognise the fact that we could have saved many lives at home and protected many others from the disease, had we carried out the measures we were able to command. Recently some have advocated the home treatment for tuberculosis, claiming that if a case is curable at all it can be cured at home; and that those who go out from these institutions much benefited stand a very poor chance of remaining so. Be this as it may, I do think that home treatment and open air treatment will, for many reasons, become warmly and closely united in the near future.

No patient, so long as he lives, should under any condition or any circumstance be excluded from fresh air and the family physician is the one to teach this. Patients can be taught at home about the dangers of the sputum, a proper receiver for it: that floors should not be carpeted and other similar precautions. Patients should be taught that a cough which is not for the purpose of expelling mucus is both useless and worthless.

### TREATMENT BY MEDICATION.

I believe that the successful treatment of this disease is often seriously retarded, if not altogether lost, in the blind belief of the efficiency of many drugs which, in the present state of our knowledge, should play a minor part, the main reliance being upon measures already enumerated. But while we hope and expect that better days will come for the unfortunate tubercular patient in the way of medication, yet no years in all the history of the past have been nearly as good as the years which have given us a very satisfactory improvement over those of bleeding, blistering, codliver oil and whiskey. The treatment by drugs since the days of the discovery of the tubercle bacillis has been one of ceaseless activity, in an attempt to either destroy the germ by germicides, or to render the soil in which they live an unfit place for their existence.

The glowing results sometimes heard of this or that drug should be received with caution. I will venture to say that no beneficial results have been obtained from any drug unless hygienic and climatic measures have been employed at the same time, and it is not safe to substitute the measures enumerated for any known drug.

In tubercular diarrhea either bismuth, or bismuth subgallate in doses of one to two drams daily, a tablet of calomel one grain and opium half a grain, often give satisfactory results: and where these fail other remedies will be equally futile in most cases. For pleuritic pains it is a good plan to immobilize the chest with adhesive strips as in fractured ribs. Creasote in full doses is still used to benefit cough and aid expectoration but no longer as a specific. A few drops of formalin on a small dish and inhaled through a long paper funnel is useful. In recent years ichthiol in capsules 15 to 50 gr. daily has largely supplanted creasote and can be used when the latter is not tolerated, and it is supposed to play a very successful warfare against the bacilli.

In hemorrhage first of all inspire confidence in the patient and thus, if possible, allay fears; rest in bed; ice to the chest; nitroglycerin, atrophia and morphia hypodermatically with liquid diet offer the best chances of relief.

A good bitter tonic about 20 minutes before meal times with pepsin after meals should generally be used. While it may be stated as a general fact that improved methods of treatment even in medication have worked wonders over our former ways, still I am sure that we are far from the ideal for which we have been searching so long and may never find. It was supposed once for a brief period, as will be remembered, that it had been found.

Sixteen years next November I happened to be in London, when Professor Koch came over from the continent, and announced his discovery, in his ever memorable address, to a large and enthusiastic gathering of the medical profession. After giving his experiences and finding that in six to eight weeks the patients were entirely free from disease he used these words as near as I can recall them: "These experiences lead me to believe that tuberculosis in the beginning can be cured with a certainty by this remedy, tuberculin." Now, the discovery of an agent which did possess such marvelous power over the tubercle bacillus and by such a renowned man of research as Koch will ever leave a wonderful page in the history of medicine.

Owing to the intense public and professional interest taken in Koch's remarkable researches and to the bitter disappointment which so soon followed the use of his tuberculin, the remembrance of that brief period will never be effaced from memory.

While the use of tuberculin is not abandoned by all at the present time yet it has not been found to do was expected of it at the time of its discovery. The caution displayed by Koch and some of his followers has not always been exhibited by some members of the medical profession, and this has done very much to bring discredit on a really meritorious remedy. This disappointment naturally renders the acceptance of the newer remedies in the same direction with an unusual amount of hesitation. Still it does seem that great progress will be made sometime in this direction, when we see the activity displayed by so many members of the medical profession working towards one result—namely, the destruction of the tubercle bacillus.

It is claimed that tuberculosis has existed since man inhabited houses. Hence, what we call our beautiful and comfortable homes the result of the architects skill and expenditure of vast sums of money, are at times the harbingers of tubercular germs which infect many individuals. The practical destruction of the North American Indian by tuberculosis alone is largely if not entirely due to his exchanging his rude out-of-door life for wretched hovels or wigwams, the best they were able to do in imitation of the houses erected by the white man. I mention this to emphasise again the importance of fresh air as obtained from out door life.

Wild animals have been inoculated with the tubercle bacillus. Divided into equal numbers, one group shut up in a close room, well fed with but little sunlight, or fresh air, invariably succumbed to the disease. The others being allowed freedom and wild life invariably recover. This is another evidence of the value of open air life.

